# The Impact of Antidiabetic Therapies on Diastolic Dysfunction and Diabetic Cardiomyopathy

**DOI:** 10.3389/fphys.2020.603247

**Published:** 2020-12-07

**Authors:** Keshav Gopal, Jadin J. Chahade, Ryekjang Kim, John R. Ussher

**Affiliations:** ^1^Faculty of Pharmacy and Pharmaceutical Sciences, University of Alberta, Edmonton, AB, Canada; ^2^Alberta Diabetes Institute, University of Alberta, Edmonton, AB, Canada; ^3^Cardiovascular Research Centre, University of Alberta, Edmonton, AB, Canada

**Keywords:** type 2 diabetes mellitus, diabetic cardiomyopathy, diastolic function, metformin, glucagon-like peptide-1 receptor agonists, dipeptidyl peptidase 4 inhibitors, sodium-glucose co-transporter 2 inhibitors

## Abstract

Diabetic cardiomyopathy is more prevalent in people with type 2 diabetes mellitus (T2DM) than previously recognized, while often being characterized by diastolic dysfunction in the absence of systolic dysfunction. This likely contributes to why heart failure with preserved ejection fraction is enriched in people with T2DM vs. heart failure with reduced ejection fraction. Due to revised mandates from major health regulatory agencies, all therapies being developed for the treatment of T2DM must now undergo rigorous assessment of their cardiovascular risk profiles prior to approval. As such, we now have data from tens of thousands of subjects with T2DM demonstrating the impact of major therapies including the sodium-glucose co-transporter 2 (SGLT2) inhibitors, glucagon-like peptide-1 receptor (GLP-1R) agonists, and dipeptidyl peptidase 4 (DPP-4) inhibitors on cardiovascular outcomes. Evidence to date suggests that both SGLT2 inhibitors and GLP-1R agonists improve cardiovascular outcomes, whereas DPP-4 inhibitors appear to be cardiovascular neutral, though evidence is lacking to determine the overall utility of these therapies on diastolic dysfunction or diabetic cardiomyopathy in subjects with T2DM. We herein will review the overall impact SLGT2 inhibitors, GLP-1R agonists, and DPP-4 inhibitors have on major parameters of diastolic function, while also highlighting the potential mechanisms of action responsible. A more complete understanding of how these therapies influence diastolic dysfunction will undoubtedly play a major role in how we manage cardiovascular disease in subjects with T2DM.

## Introduction

The rapid rise in obesity that our society has witnessed has led to an unfortunate explosion in both type 2 diabetes mellitus (T2DM) and cardiovascular disease prevalence. The most recent estimates from the International Diabetes Federation indicate that 463 million people worldwide, with 31 million adults in the United States alone, are living with diabetes in 2019, of which ~90% is accounted for by T2DM. Furthermore, the estimated global diabetes prevalence is projected to reach nearly 700 million people by 2045 (Saeedi et al., 2019). In the 1970s, reports from the Framingham Heart Study suggested that T2DM independently increases the risk of cardiovascular disease, primarily from either myocardial infarction (MI) and/or heart failure, with risk in the latter being increased 2.4-fold in men and nearly double that in women. This increased risk of heart failure is independent of associated comorbidities such as coronary artery disease, dyslipidemia, and hypertension (Kannel and Mcgee, 1979a,b). Diabetes also persists as an independent predictor of poor outcome, with diabetic subjects representing ~one-third of participants in heart failure clinical trials (Gustafsson et al., 2004; Matsue et al., 2011). Moreover, diabetes accounts for 12.8% of global all-cause mortality in adults, the majority due to cardiovascular disease (Ogurtsova et al., 2017).

Accordingly, it is imperative that we improve our understanding of the mechanisms contributing to diabetes-related heart disease, while also using clinical evidence to best guide the therapeutic choices we use to manage cardiovascular health and risk in highly susceptible diabetic individuals. Despite tight glycemic control being strongly associated with reduced risk for microvascular complications [UK Prospective Diabetes Study (UKPDS) Group, 1998b;Nathan et al., 2005], evidence to date has for most part indicated that well-managed glycemia in subjects with T2DM does not reduce the risk for macrovascular cardiovascular disease (e.g., MI, heart failure). The Action to Control Cardiovascular Risk in Diabetes (ACCORD) trial reported that intensive glucose lowering, which targeted glycated hemoglobin (HbA1c) to below 6%, increased rates of death from cardiovascular causes (Action to Control Cardiovascular Risk in Diabetes Study Group et al., 2008). Fortunately, we now have access to cardiovascular outcomes data in tens of thousands of subjects with T2DM treated with the most recent approved therapies for T2DM [sodium-glucose co-transporter 2 (SGLT2) inhibitors, glucagon-like peptide-1 receptor (GLP-1R) agonists, and dipeptidyl peptidase 4 (DPP-4) inhibitors]. The undertaking of these studies arose from the decision of major health regulatory agencies [e.g., US Food and Drug Administration (FDA)] to require pharmaceutical manufacturers of new agents for T2DM, to conduct cardiovascular outcomes trials (CVOTs) to assess the cardiovascular risk profiles of their agents compared to standard-of-care (Drucker and Goldfine, 2011).

Results from the completed and published CVOTs to date have generated much excitement in the diabetes field, which will be described throughout this review. It is important to note though that the majority of these CVOTs are investigating major adverse cardiovascular events (MACE) as a primary endpoint, which is usually comprised of death from cardiovascular causes, non-fatal MI, or non-fatal stroke, with hospitalization for heart failure as a major secondary endpoint. However, these trials are not designed to assess parameters of cardiac function in people with T2DM, or endpoints of heart failure that would be assessed in a heart failure clinical trial. The latter is of particular importance, since CVOTs do not separate individuals with T2DM who might also be comorbid for either heart failure with reduced ejection fraction (HFrEF), or heart failure with preserved ejection fraction (HFpEF). Furthermore, HFpEF is enriched in the diabetic population, and as technologies for assessing cardiac function have improved over time, it is becoming clear that diabetic cardiomyopathy, the presence of ventricular dysfunction independent of coronary artery disease and/or hypertension, is more prevalent than initially realized (Jia et al., 2018; Ritchie and Abel, 2020). As early diastolic dysfunction is often present but not diagnosed timely in people with T2DM, an area of growing importance is to better understand how current therapeutic options for people with T2DM influence diastolic function. An improved understanding in this area will have significant implications toward guiding clinical management of diabetic subjects who either have diabetic cardiomyopathy or HFpEF phenotypes. Hence, the primary aims of this review are (1) to highlight the importance of diastolic dysfunction in diabetic cardiomyopathy, and (2) to describe the impact of antidiabetic therapies including metformin, SGLT-2 inhibitors, GLP-1R agonists, and DPP-4 inhibitors on diastolic dysfunction in T2DM.

## Diabetic Cardiomyopathy

**Figure 1:**
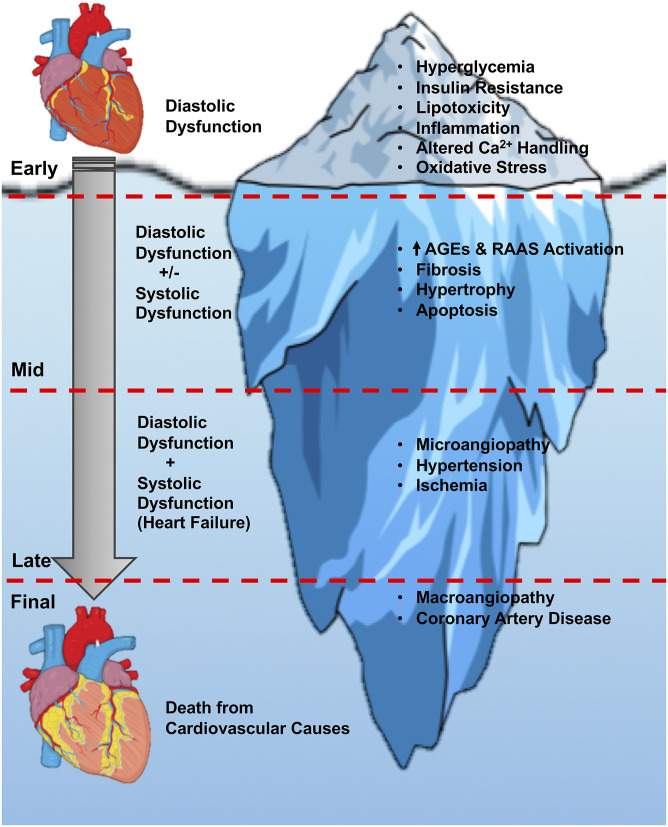
Stages of diabetic cardiomyopathy. An overview of the current understanding of the stages of diabetic cardiomyopathy progression, from the initial onset of hyperglycemia and diastolic dysfunction, through the later stages of hypertrophy, angiopathy, ischemia, and heart failure with systolic and diastolic dysfunction. The initial diastolic dysfunction that characterizes the early stages of diabetic cardiomyopathy can be viewed as just the tip of the iceberg if left untreated in people with T2DM. AGEs, advanced glycation end-products; RAAS, renin-angiotensin-aldosterone system.

As already mentioned, diabetic cardiomyopathy is the presence of ventricular dysfunction in the absence of underlying coronary artery disease and/or hypertension. It was originally described in 1972 by Rubler et al. (1972) in diabetic subjects with heart failure but no evidence of MI. In subjects with T2DM that have their blood glucose levels well managed, ~half of these individuals will exhibit some form of cardiac dysfunction even if they are clinically asymptomatic with normal blood pressure (Boyer et al., 2004; Marwick et al., 2018). Indeed, the early stages of diabetic cardiomyopathy often encompass a subclinical asymptomatic stage with increased cardiac fibrosis, reduced early diastolic filling with atrial enlargement, and an elevated left ventricular (LV) end diastolic pressure (LVEDP) (Westermeier et al., 2016). The mid-stage of the disease pathology involves cardiac remodeling, LV hypertrophy, advanced diastolic dysfunction, and the appearance of clinical indications of HFpEF, which may eventually transition into HFrEF as the disease progresses to the later stages (Figure 1) (Jia et al., 2016). Most of the advancements that have been made in understanding the mechanisms responsible for diabetic cardiomyopathy have taken place in the last 2 decades, likely influenced by major improvements in imaging technologies (e.g., ultrasound echocardiography) that have allowed more accurate assessment of diastolic function in animal studies. In contrast, the existence of diastolic dysfunction in isolation as a key feature of diabetic cardiomyopathy in humans has been challenged, often due to subjects with T2DM not undergoing rigorous assessment of diastolic function, and cardiac phenotypes only being interrogated in such individuals once overt heart failure is present (Ritchie and Abel, 2020). Nonetheless, diastolic dysfunction can be present along with insulin resistance before clinical diagnosis of T2DM (Nicolino, 1995; Di Bonito et al., 1996; Poulsen et al., 2010), and in a small study of 46 men with T2DM, was evident in 60% of subjects despite well-controlled blood glucose and without clinically detectable cardiovascular disease (Poirier et al., 2001). Moreover, in a study of 101 subjects with T2DM and no history of cardiovascular disease, diastolic dysfunction was detected in 29% of the subjects after excluding for the presence of coronary artery disease, as determined by strain analyses and peak diastolic velocity measurements (Fang et al., 2005). In the following section, we will highlight the major proposed mediators of diabetic cardiomyopathy, while detailing the primary parameters of diastolic function that are commonly assessed in studies of diabetic cardiomyopathy.

### Mediators of Diabetic Cardiomyopathy

The majority of proposed disease mechanisms responsible for diabetic cardiomyopathy have been uncovered using animal models of obesity/insulin resistance (diet-induced or genetically mediated) that attempt to mimic the pathophysiology commonly observed in diabetic humans. A number of different animal models are available for the study of diabetic cardiomyopathy, and the strengths and weaknesses of these various models have been extensively reviewed elsewhere (Bugger and Abel, 2009; Riehle and Bauersachs, 2018). However, with relation to T2DM, the vast majority of studies use either leptin receptor deficient *db*/*db* mice, or mice subjected to obesity via high-fat diet (HFD) supplementation combined with a low-dose injection with the β-cell toxin, streptozotocin (STZ). Although these 2 models do not perfectly reproduce the human condition, they do share key features of human diabetic cardiomyopathy, including cardiac fibrosis and early diastolic dysfunction in the absence of overt systolic dysfunction (Ritchie and Abel, 2020). Common mechanisms precipitating diabetic cardiomyopathy include systemic and cardiac insulin resistance, altered myocardial substrate utilization, and mitochondrial dysfunction, all of which can increases oxidative stress in the diabetic myocardium. Furthermore, hyperglycemia and glucotoxicity, myocardial lipid accumulation and lipotoxicity, inflammation, endoplasmic reticulum stress, cardiac fibrosis, microvascular dysfunction, elevations in intracellular Na^+^, and impaired myocardial Ca^2+^ signaling/handling are also all implicated in mediating diabetic cardiomyopathy, though we encourage the reader to refer to the many excellent reviews on this topic for in-depth information (Battiprolu et al., 2010; Van De Weijer et al., 2011; D'souza et al., 2016; Zlobine et al., 2016; Jia et al., 2018; Ritchie and Abel, 2020). Although it remains challenging to determine how each of these factors may individually contribute to diastolic dysfunction in people with T2DM, all of these various mediators do appear capable of influencing one another through various cross-talk mechanisms (Jia et al., 2018; Ritchie and Abel, 2020). It also remains unknown which of these potential mediators will prove most useful as a target for improving diastolic dysfunction, and thus future preclinical studies aimed at understanding the sequential nature of these mechanisms in subjects with T2DM is necessary.

### Diastolic Dysfunction and its Assessment in Diabetic Cardiomyopathy

While the gold-standard procedure for assessing cardiac function in either clinical or preclinical studies is largely considered to be cardiac magnetic resonance (CMR) imaging, ultrasound echocardiography is usually more readily available and has underwent a series of major advancements (e.g., Tissue Doppler imaging) that have improved its ability to assess diastolic function (Ho and Solomon, 2006; Lindsey et al., 2018). Therefore, recent guidelines published by the American Society of Echocardiography and the European Association of Cardiovascular Imaging, provide guidance on a series of Tissue Doppler-based signals acquired by ultrasound echocardiography for assessing diastolic function (Nagueh et al., 2016). Common parameters of assessment include Doppler echocardiography to assess the relationship of peak velocity blood flow during LV relaxation in early diastole (E wave) to peak velocity blood flow in late diastole caused by atrial contraction (A wave), denoted as the mitral E/A ratio, which often decreases with diastolic dysfunction (Ho and Solomon, 2006; Ritchie and Abel, 2020). Similarly, by using Tissue Doppler imaging one can assess velocity of the adjacent myocardial tissue during both the early (e′) and late (a′) phases of diastole, with the e′/a′ ratio also frequently decreasing in the presence of diastolic dysfunction. A very reliable and reproducible measure of diastolic dysfunction involves both Doppler echocardiography and Tissue Doppler imaging to quantify the E/e′ ratio, which increases with diastolic dysfunction (Ritchie and Abel, 2020). In addition, measuring global diastolic strain rate is also useful in the assessment of diastolic function, as it is independent of load and accounts for LV size (Wang et al., 2007). On the contrary, diastolic function can also be inferred from the isovolumic ventricular relaxation time (IVRT), but this parameter is highly dependent on load and heart rate (Nagueh et al., 2016). Although these echocardiographic parameters can be used to estimate LVEDP and overall diastolic function in a population-based statistical sense, one must still exercise caution when assessing these parameters in an individual patient and account for other influencing factors (e.g., age, sex, overall patient history, etc.) (Mitter et al., 2017).

## Pharmacotherapies for T2DM and Their Impact On the Pathogenesis of Diabetic Cardiomyopathy

Clinicians currently have at their disposal a wide armamentarium of therapies targeting differing mechanisms of actions for improving glycemia in people with T2DM. This includes the first-line therapy, metformin, as well as other oral therapies including sulfonylureas, thiazolidinediones, and the α-glucosidase inhibitors. The twenty-first century has seen the approval of a number of new drug classes for treating T2DM, including the SGLT2 inhibitors, as well as the GLP-1R agonists and DPP-4 inhibitors, often collectively referred to as incretin-based therapies. Despite metformin being generally recommended as the first-line treatment, control of HbA1c levels is for most part consistent amongst the majority of approved medications, though most individuals with T2DM will eventually require combination therapy to adequately control their hyperglycemia (Qaseem et al., 2012). As the majority of older pharmacotherapies for T2DM were not subjected to extensive assessment of cardiovascular risk by the major health regulatory agencies, we will focus primarily on the impact the twenty-first century therapies potentially have on diabetic cardiomyopathy, due to the plethora of available data from CVOTs. Nevertheless, as metformin is generally considered to be either cardiovascular neutral or cardioprotective (Driver et al., 2018; Rena and Lang, 2018), while being the first-line medication for T2DM, we will also provide an overview of metformin's impact on the pathology of diabetic cardiomyopathy for comparison. The reader is encouraged to read other reviews (Riveline et al., 2003; Loke et al., 2011; Ussher et al., 2012; Herman et al., 2017) that have already described the cardiovascular actions in-depth for the antidiabetic therapies not covered herein (e.g., sulfonylureas, thiazolidinediones, α-glucosidase inhibitors).

## Metformin

Metformin is an oral anti-diabetic agent belonging to the biguanide drug class and is the first-line therapy of choice in the management of glycemia in patients with T2DM (American Diabetes Association, 2009). Metformin's mechanism of action is generally attributed to the activation of 5-AMP activated protein kinase (AMPK). Increased AMPK activity reduces the expression of genes involved in the regulation of hepatic gluconeogenesis, phosphoenolpyruvate carboxykinase, and glucose-6-phosphatase, thereby accounting for the marked reduction in hepatic glucose production (HGP) observed in humans treated with metformin (Kim et al., 2008). Of relevance, metformin continues to have its mechanism of action further scrutinized, with recent studies indicating that the role of AMPK in mediating metformin's ability to reduce HGP involves inhibition of acetyl CoA carboxylase (Fullerton et al., 2013). This relieves malonyl CoA-induced inhibition of fatty acid oxidation, which lowers hepatic lipid accumulation and subsequent HGP (Fullerton et al., 2013). Conversely, it has also been demonstrated that metformin may reduce HGP via suppression of glucagon mediating signaling in the liver (Miller et al., 2013), whereas other studies have demonstrated that metformin inhibits the mitochondrial glycerophosphate dehydrogenase to alter redox state and decrease HGP (Madiraju et al., 2014). With regards to metformin's actions on the cardiovascular system, metformin was initially contraindicated in patients with heart failure due to its potential to increase lactic acidosis (Ussher et al., 2012). However, in 2006 the US FDA removed the heart failure contraindication from metformin's product label, and a systematic review in patients with T2DM and heart failure concluded that metformin was the only antidiabetic agent not associated with an increase in harm, though 7 of the 8 studies included were observational (Eurich et al., 2007). Furthermore, analysis of metformin use in the UK Prospective Diabetes Study in 4,075 individuals with newly diagnosed T2DM demonstrated that metformin use resulted in a 30% lower risk than conventional treatment for macrovascular disease (MI, sudden death, angina, stroke, and peripheral disease) [UK Prospective Diabetes Study (UKPDS) Group, 1998a]. Recent studies in animals and humans have also demonstrated favorable cardioprotective properties of metformin. Hence, a reappraisal of metformin's impact on cardiovascular outcomes in comparison to that of GLP-1R agonists, DPP-4 inhibitors, and SGLT2 inhibitors should be taken into consideration with regards to how we manage people with T2DM.

### Metformin and Diabetic Cardiomyopathy in Humans

Despite being one of the antidiabetic therapies not subject to extensive interrogation relating to cardiovascular outcomes, metformin's impact on cardiac function has been investigated in a number of human studies. A prospective study in 1,519 patients with heart failure and new-onset T2DM observed that treatment with metformin resulted in a decrease in mortality, primarily due to a reduction in cardiovascular mortality (Romero et al., 2011). Consistent with lactic acidosis risk being considerably lower in patients receiving metformin vs. previous biguanide agents (phenformin), no case of lactic acidosis was reported in this study. Moreover, an observational study in 422 patients with T2DM and chronic heart failure demonstrated that both metformin monotherapy or metformin plus sulfonylurea therapy resulted in a reduced risk for all-cause mortality during 1-year and long-term follow-up vs. sulfonylurea therapy alone (Evans et al., 2010). A meta-analysis of 2,079 individuals with T2DM across 13 trials between 1995 to 2011 demonstrated that all-cause mortality, cardiovascular death, MI, and peripheral disease all favored metformin use (vs. either no intervention, placebo, or lifestyle intervention), but none of these observations reached statistical significance (Griffin et al., 2017). Regarding diastolic dysfunction, compared to sulfonylurea or insulin therapy, metformin was associated with improved diastolic function as indicated by a lower IVRT in an observational study of 242 diabetic patients undergoing coronary angiography (Andersson et al., 2010). Furthermore, the METformin in DIastolic Dysfunction of MEtabolic syndrome (MET-DIME) trial is a phase II prospective and randomized blinded, open-label trial investigating the impact of metformin treatment (target dose 1,000 mg twice daily) with a 2-year follow up in 54 individuals with diastolic dysfunction (Ladeiras-Lopes et al., 2014). The primary endpoint being measured is e′, with secondary endpoints including other parameters of diastolic function (e.g., E/A ratio, IVRT, etc.). Of interest, initial findings at 1-year of follow-up have demonstrated that metformin treatment does appear to improve diastolic function, as reflected by differences in e′ (−0.52 cm/s in the controls vs. +0.46 cm/s with metformin) (Ladeiras-Lopes et al., 2019).

### Metformin and Diabetic Cardiomyopathy in Preclinical Studies

Metformin has well-documented actions supporting cardioprotective actions of this medication in preclinical models of ischemic heart disease and heart failure (extensively reviewed in Ussher et al., 2012). With regards to diabetic cardiomyopathy and diastolic function, metformin does appear to have salutary actions in experimental diabetic cardiomyopathy, potentially through activation of AMPK-dependent cardiac myocyte autophagy (Xie et al., 2011). OVE26 mice are a model of early-onset T1D due to calmodulin overexpression in islet β-cells, and metformin treatment for 4-months (200 mg/kg in the drinking water) improved indices of both systolic (e.g., LVEF) and diastolic (e.g., mitral E/A ratio) function. In male C57BL/6J mice subjected to experimental T1D via 5 consecutive daily intraperitoneal injections with streptozotocin (STZ; 50 mg/kg), metformin treatment for 16-weeks (250 mg/kg in the drinking water) improved systolic function (e.g., LVEF) and cardiac hypertrophy (Yang et al., 2020). Although parameters of diastolic function were not assessed in this study, metformin treatment also reduced cardiac fibrosis (key mediator of diabetic cardiomyopathy) as indicated by less collagen deposition in Masson's trichrome stained LV cross-sections. Furthermore, spontaneously hypertensive rats, which develop obesity and insulin resistance, exhibit significant improvements in LVEF, cardiac hypertrophy, and end-diastolic wall thickness following 3-months of metformin treatment (300 mg/kg in the drinking water). Taken together, metformin does appear to have salutary effects on cardiac function in T2DM, both in clinical and preclinical studies. However, there has been limited overall evaluation of diastolic function in these studies, and thus well-designed studies in animal models of T2DM that exhibit diastolic dysfunction and cardiomyopathy phenotypes in the absence of overt systolic dysfunction are needed.

## Sodium-Glucose Co-Transporter 2 Inhibitors

SGLT2 is found almost exclusively in the proximal convoluted tubules of nephrons, and is responsible for reabsorbing ~97% of renally filtered glucose (Heerspink et al., 2016). Accordingly, the SGLT2 inhibitors (e.g., empagliflozin, canagliflozin, dapagliflozin), were developed as potential antidiabetic agents that promote glucose lowering by blocking the reabsorption of renally filtered glucose, which is subsequently excreted in the urine (glucosuria). In practice, treatment with SGLT2 inhibitors only prevents ~50–60% of glucose reabsorption, due to increased SGLT1 mediated glucose reabsorption in the later sections of the proximal convoluted tubule, for which these agents have limited selectivity towards (Rieg et al., 2014; Heerspink et al., 2016). SGLT2 inhibitors differ from the majority of antidiabetic agents, as they facilitate the elimination of glucose rather than stimulate its uptake via either increasing insulin secretion or insulin sensitivity. Due to this unique mechanism of action, SGLT2 inhibitors have a significantly lower risk of hypoglycemia, while lowering HbA1c by comparable levels (Heerspink et al., 2016). Importantly, since SGLT2 inhibitors achieve their glucose-lowering effect through an insulin-independent mechanism, they permit lower insulin requirements of subjects with T2DM and can be used effectively in any stage of T2DM (Vallon, 2015). As a result of their safety profile, reasonable efficacy, and cardiovascular benefits that will be described in the following section, SGLT2 inhibitors have found increasing utilization in clinical practice guidelines (Davies et al., 2018; Diabetes Canada Clinical Practice Guidelines Expert Committee et al., 2018). Because SGLT2 does not appear to be expressed in cardiac myocytes (Chen et al., 2010), there has also been increasing scrutiny to elucidate the mechanisms by which SGLT2 inhibitors induce cardioprotection.

### SGLT2 Inhibitors and Cardiovascular Outcomes

Large scale CVOTs involving SGLT2 inhibitors have consistently demonstrated cardiovascular benefit in people with T2DM with regards to reducing 3-point MACE. Excitement in this field was first engendered following completion of the Empagliflozin Cardiovascular Outcome Event Trial in T2DM Patients (EMPA-REG) (Zinman et al., 2015), which reported significantly reduced rates for 3-point MACE in patients assigned empagliflozin compared to standard of care (37.4 vs. 43.9 events per 1,000 patient-years). In addition, hospitalization for heart failure, a secondary endpoint of the trial, was also lower in patients assigned empagliflozin (35% relative risk reduction). Similarly, the Canagliflozin Cardiovascular Assessment Study (CANVAS), an integration of results from the CANVAS and the CANVAS-Renal trials, also reported fewer events in subjects treated with canagliflozin using 3-point MACE (26.9 vs. 31.5 events per 1,000 patient-years) (Neal et al., 2017). Conversely, the Dapagliflozin Effect on CardiovascuLAR Events (DECLARE-TIMI 58) trial did not observe a reduction in events using 3-point MACE in subjects treated with dapagliflozin (Wiviott et al., 2019). Importantly, all 3 of the EMPA-REG, CANVAS, and DECLARE-TIMI 58 trials reported significantly fewer hospitalizations for heart failure. Although it remains unknown why 3-point MACE was not improved with dapagliflozin, subjects with chronic kidney disease appear to have greater benefits with SGLT2 inhibitors than other populations (Bloomgarden, 2018), and the DECLARE-TIMI 58 trial did include more restrictive creatinine clearance exclusion criteria (<60 mL/min vs. <30 mL/min in both EMPA-REG and CANVAS). Taken together, SGLT2 inhibitors have consistently improved cardiovascular outcomes in people with T2DM, especially with regards to reducing hospitalization rates for heart failure. In further support of this, the Dapagliflozin and Prevention of Adverse Outcomes in Heart Failure (DAPA-HF) trial examined 4,744 subjects with pre-existing heart failure (LVEF <40%) followed for a median time of 18.2 months assigned to either dapagliflozin (10 mg once daily) or placebo, and reported fewer events involving hospitalization for heart failure, urgent heart-failure visit, and cardiovascular death (Mcmurray et al., 2019). Interestingly, these observations were seen both in the subjects with and without T2DM. Although there is evidence to suggest that SGLT2 inhibitors do benefit individuals with HFrEF, none of the aforementioned studies were specifically designed to evaluate diastolic function or HFpEF, which is highly relevant when considering the impact of this drug-class on T2DM subjects with diabetic cardiomyopathy.

### SGLT2 Inhibitors and Diabetic Cardiomyopathy in Humans

Large scale clinical trials specifically investigating the impact of SGLT2 inhibitors on diastolic dysfunction/diabetic cardiomyopathy are lacking. Nonetheless, emerging evidence from smaller trials have examined specific parameters of diastolic function and noted salutary actions in response to SGLT2 inhibition (Table 1). A *post-hoc* analysis of transthoracic echocardiograms from 10 subjects in the EMPA-REG OUTCOME trial observed a 15% decrease in the LV mass index (LVMI), and an increase in the e′ in subjects treated with empagliflozin for ~3 months (Verma et al., 2016). Likewise, CMR imaging in 25 subjects with T2DM demonstrated that empagliflozin treatment for 6 months (10 mg once daily) lowered LV end diastolic volume compared to control subjects (−15.4 mL mean difference) (Cohen et al., 2019). Although SGLT2 does not appear to be expressed in cardiac myocytes (Chen et al., 2010), studies in single de-membranated cardiac myocytes from humans with HFpEF demonstrated that treatment with empagliflozin (0.5 μM) decreases myofilament passive stiffness, which may contribute to empagliflozin mediated improvements in diastolic function (Pabel et al., 2018). Similarly, in 37 subjects with T2DM, 32% of which had pre-existing cardiovascular disease, a 3-month treatment with canagliflozin (100 mg once daily) decreased both the E/e′ (12%) and LVMI (6%) (Matsutani et al., 2018), whereas dapagliflozin treatment for 6-months in 57 subjects with T2DM and stable heart failure also decreased both the E/e′ (9%) and LVMI (11%) (Soga et al., 2018). A *post-hoc* analysis of the latter study reported that dapagliflozin treatment also improved global longitudinal strain (1.4% increase), a parameter that is used to predict subclinical LV dysfunction and for identifying individuals more likely to progress to heart failure (Tanaka et al., 2020). Moreover, the impact of dapagliflozin on improving global longitudinal strain was greater in individuals with HFpEF vs. those with HFrEF. Last, in an observational study of 26 subjects with T2DM treated for ~8-months with tofogliflozin (20 mg once daily), an SGLT2 inhibitor approved in Japan, an improvement in diastolic function as indicated by a lowering of the E/e′ was observed compared to 162 subjects with T2DM treated with other glucose-lowering medications (Otagaki et al., 2019). While the majority of human studies assessing the impact of SGLT2 inhibitors on diastolic function are limited by their overall sample sizes and reduced treatment durations when compared to their respective CVOTs, there is a common trend among all approved SGLT2 inhibitors of lowering E/e′ and LVMI. The extent to which these improvements in diastolic function account for the benefit seen in large scale CVOTs for SGLT2 inhibitors remains to be determined. Thus, both larger human studies and mechanism-focused preclinical studies, are needed to further understand the place SGLT2 inhibitors have in managing diabetic cardiomyopathy and diabetes-related cardiovascular disease.

**Table 1:** The impact of SGLT2 inhibitors on diabetic cardiomyopathy.

**SGLT2 inhibitor**	**Study model**	**SGLT2 inhibitor mediated outcome**	**References**
Empagliflozin (10 mg once daily)	Subjects with T2DM and history of cardiovascular disease treated with empagliflozin for 3-months	• Decreased LVMI increased e′	Verma et al., 2016
Empagliflozin (10 mg once daily)	Subjects with T2DM (20% with cardiovascular disease) treated with empagliflozin for 6-months	• Decreased LV end diastolic volume	Cohen et al., 2019
Canagliflozin (100 mg once daily)	Subjects with T2DM (32% with cardiovascular disease) treated with canagliflozin for 3-months	• Decreased LVMI • Decreased E/e′ ratio	Matsutani et al., 2018
Dapagliflozin (5 mg once daily)	Subjects with T2DM and stable heart failure treated with dapagliflozin for 6-months	• Decreased LVMI • Decreased E/e′ ratio • Increased global Longitudinal strain	Soga et al., 2018; Tanaka et al., 2020
Tofogliflozin (20 mg once daily)	Subjects with T2DM without cardiovascular disease treated with tofogliflozin for ~8-months	• Decreased E/e′ ratio	Otagaki et al., 2019
Empagliflozin (60 mg per kg of chow)	Female *db/db* mice treated with empagliflozin for 5-weeks	• Decreased E/e′ ratio • Increased e′/a′ ratio	Habibi et al., 2017
Empagliflozin (25 mg/kg intravenously)	ZDF rats treated acutely with empagliflozin	• Decreased IVRT • Increased mitral E/A ratio	Pabel et al., 2018
Empagliflozin (10 mg/kg/day)	Male *db/db* mice treated with empagliflozin for 8-weeks	• Decreased IVRT • Increased mitral E/A ratio	Xue et al., 2019
Empagliflozin (10 mg/kg/day)	Male Dahl salt-sensitive rats fed a high-salt diet and treated with empagliflozin for 2-weeks	• Decreased E/e′ ratio	Byrne et al., 2020
Empagliflozin (10 mg/kg/day in the chow)	Male *db/db* mice treated with empagliflozin for 4-weeks	• No effect on the E/e′ ratio	Verma et al., 2018

### SGLT2 Inhibitors and Diabetic Cardiomyopathy in Preclinical Studies

The SGLT2 inhibitor mediated alleviation of diastolic dysfunction has been recapitulated in several preclinical studies of either experimental T2DM or heart failure, especially with regards to empagliflozin (Table 1). For example, empagliflozin treatment (10 mg/kg once daily) for 5-weeks in 11-week-old female *db*/*db* mice improved diastolic function as indicated by a lowering of the E/e′ and increase in the e′/a′ ratios, without impacting parameters of systolic function (Habibi et al., 2017). Furthermore, intravenous treatment of empagliflozin (25 mg/kg) lowered the IVRT and increased the mitral E/A ratio without impacting systolic function in Zucker diabetic fatty (ZDF) rats following just 30 min of administration (Pabel et al., 2018). Similarly, empagliflozin treatment (10 mg/kg) for 8-weeks lowered the IVRT and increased the mitral E/A ratio, while also improving systolic function in 7-week-old male *db*/*db* mice (Xue et al., 2019). These salutary actions extend to HFpEF independent of T2DM, as treatment with empagliflozin (10 mg/kg once daily via oral gavage) for 2-weeks also lowered the E/e′ ratio in male Dahl salt-sensitive rats that were supplemented with 8.0% salt in their diet to induce experimental HFpEF (Byrne et al., 2020). In contrast, empagliflozin treatment (10 mg/kg/day in the diet) for 4-weeks in 18-week-old male *db*/*db* mice had no effect on the E/e′ ratio, though it should be noted that diastolic dysfunction was not present in these *db*/*db* mice when compared to their lean C57BL/6J controls (Verma et al., 2018).

Postulated mechanisms for the improvement in diastolic function following treatment with empagliflozin in experimental T2DM include potential increases in myocardial ketone metabolism, as use of SGLT2 inhibitors leads to increases in circulating ketones, which are a more efficient fuel than fatty acids (Ferrannini et al., 2016; Lopaschuk and Verma, 2016). However, isolated working hearts from 18-week-old male *db*/*db* mice provided empagliflozin in their diet for 4-weeks at an estimated daily dose of 10 mg/kg, do not exhibit an increase in ketone oxidation (Verma et al., 2018). Likewise, this has been recapitulated using *in vivo* hyperpolarized ^13^C magnetic resonance spectroscopy in 10-week-old male obese spontaneously hypertensive, heart failure prone rats treated for 6-months with empagliflozin (25 mg/kg once daily via oral gavage) (Abdurrachim et al., 2019). Instead, SGLT2 inhibitor mediated increases in circulating ketones may improve diastolic function by reducing activation of the nucleotide-binding domain-like receptor protein 3 regulated inflammasome and subsequent cardiac inflammation (Ye et al., 2017; Xue et al., 2019; Byrne et al., 2020). As previously described, increases in cardiac fibrosis are key contributors to increased myocardial stiffness and diastolic dysfunction (Jia et al., 2018; Ritchie and Abel, 2020), and treatment with empagliflozin is often associated with decreased myocardial mRNA expression of gene markers of fibrosis and subsequent collagen deposition (Habibi et al., 2017; Xue et al., 2019). Intriguingly, impaired soluble guanylate cyclase (sGC) mediated activation of protein kinase G signaling may be a key molecular regulator of diastolic dysfunction (Van Heerebeek et al., 2012), and the empagliflozin mediated alleviation of diastolic dysfunction in *db*/*db* mice is mitigated via intravenous siRNA delivery to decrease myocardial sGC-β expression (Xue et al., 2019). It has also been demonstrated that the SGLT2 inhibitors empagliflozin and dapagliflozin have direct actions on human coronary artery endothelial cells, as both SGLT2 inhibitors at 1 μM prevented the increase and decrease in reactive oxygen species and nitric oxide levels, respectively, in response to treatment with 10 ng/mL tumor necrosis factor-α (TNFα) (Uthman et al., 2019). Furthermore, treatment of cardiac microvascular endothelial cells with 1 μM empagliflozin prevented the adverse effects of TNFα (10 ng/mL) on nitric oxide bioavailability and contractility/relaxation of co-cultured adult cardiac myocytes (Juni et al., 2019). Given the role of nitric oxide in regulating sGC mediated signaling in the vasculature, it may suggest that SGLT2 inhibitors also alleviate diabetic cardiomyopathy via improving microvascular function.

Because SGLT2 is not expressed in cardiac myocytes, it is likely that these signaling mechanisms are mediated via indirect actions on other tissues or the coronary vasculature as just described above. Conversely, the immediate improvement following 30 min of treatment with empagliflozin to improve diastolic function in ZDF rats (Pabel et al., 2018) suggests that direct cardiac actions may still be involved. As such, it remains possible that direct cardiac actions mediated via SGLT2 inhibitors to improve diastolic dysfunction in diabetic cardiomyopathy are the result of off-target actions. Indeed, empagliflozin has been shown to directly inhibit the sodium/hydrogen exchanger (NHE) in mice and rabbit cardiac myocytes within 10 min of exposure at a 1 μM concentration, to ~80% of the reduction seen following treatment with the NHE inhibitor cariporide (Baartscheer et al., 2017). In follow-up studies from these same investigators, *in-situ* molecular modeling demonstrated that empagliflozin, dapagliflozin, and canagliflozin all favorably bind the NHE at the extracellular Na^+^-binding site, leading to an inhibition of NHE activity (Uthman et al., 2018). These findings have also recently been recapitulated in human atrial cardiac myocytes exposed to 1 μM empagliflozin, with the level of NHE inhibition being comparable to that observed with 10 μM cariporide (Trum et al., 2020). Taken together, the completed preclinical studies to date suggest that SGLT2 inhibitors positively influence multiple factors strongly associated with improvements in diastolic function in T2DM, and possibly through a combination of indirect on-target and direct off-target effects.

## Glucagon-Like Peptide-1 Receptor Agonists

GLP-1 is an incretin hormone synthesized and secreted from enteroendocrine L-cells primarily in the small intestine in response to nutrient ingestion, though it is also produced by preproglucagon expressing neurons in the brain (Campbell and Drucker, 2013). The biological actions of GLP-1 are mediated through transducing the actions of the GLP-1R, a G-protein coupled receptor belonging to the Class B Family of G-protein coupled receptors. The GLP-1R was originally identified in the pancreas but is also widely expressed in extrapancreatic tissues such as the lungs, enteric nervous system, atrial myocardium, vascular smooth muscle cells, and regions of the brain (Campbell and Drucker, 2013). Of importance to glycemic control in T2DM, GLP-1 acts on the islet β-cell GLP-1R to potentiate insulin secretion in a glucose-dependent manner, greatly reducing the risk for hypoglycemia compared to other insulin secretagogues. These agents have made a major impact in managing T2DM, as in addition to potentiating insulin secretion, they have numerous other pleiotropic effects that benefit subjects with T2DM. This includes reductions in appetite that frequently lead to body weight loss, as well as reducing glucagon secretion and gastric emptying, all of which contribute to the glucose-lowering actions attributed to GLP-1R agonists (Campbell and Drucker, 2013). Finally, results from large scale CVOTs have now revealed that many but not all GLP-1R agonists yield significant improvements in cardiovascular health in people with T2DM, suggesting that use of these agents is likely to increase due to the importance of managing cardiovascular risk in these individuals.

### GLP-1R Agonists and Cardiovascular Outcomes

The Evaluation of Lixisenatide in Acute Coronary Syndrome (ELIXA) trial demonstrated that treatment of subjects with T2DM with lixisenatide was non-inferior to placebo for 3-point MACE and hospitalization for heart failure (Pfeffer et al., 2015). Similarly, rates of 3-point MACE and hospitalization for heart failure were not different vs. placebo with once weekly exenatide treatment in subjects with T2DM in the Exenatide Study of Cardiovascular Event Lowering (EXSCEL) trial (Holman et al., 2017). While the results of these CVOTs were not met with major excitement from a cardiovascular perspective, completed CVOTs for other GLP-1R agonists have been quite positive. The Liraglutide Effect and Action in Diabetes: Evaluation of Cardiovascular Outcome Results (LEADER), the Trial to Evaluate Cardiovascular and Other Long-term Outcomes with Semaglutide (SUSTAIN-6), the Albiglutide and Cardiovascular Outcomes in Patients with T2D and Cardiovascular Disease (HARMONY Outcomes), and the Researching Cardiovascular Events With a Weekly Incretin in Diabetes (REWIND) CVOTs all resulted in significant reductions for 3-point MACE (Marso et al., 2016a,b; Hernandez et al., 2018; Gerstein et al., 2019). Of interest, reductions in non-fatal stroke were the primary driver of the improved cardiovascular outcomes in both SUSTAIN-6 and REWIND, whereas reductions in MI contributed to the improved cardiovascular outcomes in LEADER and HARMONY Outcomes. Findings among these 4 CVOTs were not entirely consistent though with respect to rates of hospitalization for heart failure. While LEADER reported a trend toward reduced rates of hospitalization for heart failure (*P* = 0.14) with liraglutide treatment, both SUSTAIN 6 and REWIND reported no difference with semaglutide or dulaglutide treatment, respectively, whereas a composite of death from cardiovascular causes or hospitalization for heart failure also revealed a trend to improvement with albiglutide treatment (*P* = 0.11) in HARMONY Outcomes.

As previously emphasized, all the completed large-scale CVOTs to date were not designed to specifically test for efficacy in people with T2DM and HFrEF vs. T2DM and HFpEF, or how they simply impact cardiac function in HFrEF or HFpEF independent of T2DM. Findings from a small non-randomized study of 21 subjects with class New York Heart Association class III/IV heart failure and LVEF ≤ 40%, demonstrated that a 5-week GLP-1 infusion (2.5 pmol/kg/min) improved LVEF and 6-min treadmill walking distance (Sokos et al., 2006). Conversely, the phase 2 Functional Impact of GLP-1 for Heart Failure Treatment (FIGHT) study demonstrated that liraglutide treatment (uptitrated to 1.8 mg once daily) over 180 days in subjects with severe heart failure (LVEF ≤40%) resulted in a numerical increase in rates of death or rehospitalization for heart failure (*P* = 0.14) (Margulies et al., 2016). This trend was stronger in subjects with T2DM (*P* = 0.07), but treatment with liraglutide did not adversely affect secondary heart failure endpoints including the change from baseline for both LVEF and 6-min walk distances. Reasons for the discrepancies between these 2 studies of heart failure subjects are unknown but could involve differences in treatment duration, the fact that the former study was open-label and non-randomized, as well as the type of GLP-1R agonist utilized. The former study used native GLP-1 infusions to systemically activate the GLP-1R, and it is worth noting that the DPP-4 mediated GLP-1 breakdown product, GLP-1(9-36), may produce cardioprotective actions that would not be present with many GLP-1R agonists (Ussher and Drucker, 2014).

### GLP-1R Agonists and Diabetic Cardiomyopathy in Humans

With regards to HFpEF or diastolic dysfunction in subjects with T2DM, a number of small-scale trials have reported salutary actions of GLP-1R agonists on diastolic function (Table 2). The MAGNetic resonance Assessment of VICTOza efficacy in the Regression of cardiovascular dysfunction In type 2 dIAbetes mellitus (MAGNA VICTORIA) study was a single center trial in 49 T2DM subjects without cardiovascular disease randomized to treatment with liraglutide (uptitrated to 1.8 mg once daily) or placebo (Bizino et al., 2019). Treatment with liraglutide for 6-months improved diastolic function, as indicated by an increased e′ and decreased LV end diastolic volume, which resulted in a trend toward an improved LV compliance. Similarly, in a small observational study of 37 subjects with T2DM and no history of acute coronary disease, treatment with liraglutide for 6-months (uptitrated to 1.8 mg once daily) also improved diastolic function as seen by an increased e′ and e′/a′ ratio, and a reduced E/e′ ratio. The improvement in diastolic function occurred independent of changes in parameters of systolic function, but it should be noted that the control subjects in this study were individuals who discontinued liraglutide therapy after 1 month and were switched to a different glucose-lowering medication (Saponaro et al., 2016). Furthermore, decreases in both pulse wave velocity and the E/e′ ratio, indicative of improved arterial stiffness and diastolic function, respectively, were reported in 11 subjects with T2DM treated with exenatide (10 μg twice daily) over 3-months vs. 12 placebo treated subjects (Scalzo et al., 2017). In contrast, no differences in diastolic function using CMR imaging to assess the circumferential peak early diastolic strain rate were observed following treatment with either liraglutide (uptitrated to 1.8 mg once daily) or sitagliptin (100 mg once daily) for 6-months in the impact of Liraglutide on cardiac function and structure in Young adults with type 2 DIAbetes (LYDIA) study (Webb et al., 2020). Although no changes in diastolic function were detected between the two treatment arms, one must be cautious in drawing firm conclusions regarding liraglutide's impact on diastolic function in this study, due to the lack of a placebo control arm in subjects with T2DM.

**Table 2:** The impact of GLP-1R agonists on diabetic cardiomyopathy.

**GLP-1R agonist**	**Study model**	**GLP-1R agonist mediated outcome**	**References**
Liraglutide (uptitrated to 1.8 mg once daily)	Subjects with T2DM and no history of cardiovascular disease treated with liraglutide for 6-months	• Increased e′ • Decreased LV end diastolic volume	Bizino et al., 2019
Liraglutide (uptitrated to 1.8 mg once daily)	Subjects with T2DM and no history of acute coronary disease treated with liraglutide for 6-months	• Increased e′ • Increased e′/a′ ratio • Decreased E/e′ ratio	Saponaro et al., 2016
Exenatide (10 μg twice daily)	Subjects with T2DM treated with exenatide for 3-months	• Decreased pulse wave velocity • Decreased E/e′ ratio	Scalzo et al., 2017
Liraglutide (uptitrated to 1.8 mg once daily)	Subjects with T2DM and 65% having ≥1 cardiovascular risk factor treated with liraglutide for 6-months	• No difference in circumferential peak early diastolic strain rate	Webb et al., 2020
Liraglutide (30 μg once daily)	Male C57BL/6J mice subjected to T2DM and TAC surgery, treated with liraglutide for 15-weeks	• Liraglutide treatment prevented a decline in the LVEF in response to TAC	Mulvihill et al., 2016
Liraglutide (30 μg twice daily)	Male C57BL/6J mice subjected to T2DM and treated with liraglutide for 2-weeks	• Increased mitral E/A ratio • Decreased E/e′ ratio	Almutairi et al., 2020
Exendin-4 (100 μg/kg once daily SQ)	Male C57BL/6 mice subjected to T2DM and treated with exendin-4 for 8-weeks	• Increased mitral E/A ratio • Increased LVEF	Wu et al., 2018
Liraglutide (75 μg once daily)	Male Sprague-Dawley rats subjected to T2DM treated with liraglutide for 4-weeks.	• Decreased oxidative stress and cardiac myocyte apoptosis	Hussein et al., 2020
Exenatide (24 nmol/kg once daily IP)	Male C57BL/6J mice subjected to experimental obesity and treated with exenatide for 4-weeks.	• Decreased oxidative stress • Increased mitral E/A ratio	Ding et al., 2019

### GLP-1R Agonists and Diabetic Cardiomyopathy in Preclinical Studies

Recapitulating the observations reported in humans with T2DM, preclinical studies have also revealed that GLP-1R agonists induce robust cardioprotection (extensively reviewed in Ussher and Drucker, 2014; Drucker, 2016), actions that appear to extend to experimental diabetic cardiomyopathy (Table 2). In male C57BL/6J mice subjected to T2DM via HFD supplementation (45% kcal from lard) for 28-weeks with STZ (90 mg/kg) administered at the 12-week time point, treatment with liraglutide for the final 15-weeks (30 μg/kg once daily) prevented a decline in LVEF in response to transverse aortic constriction (TAC) surgery performed at the 18-week time point (Mulvihill et al., 2016). However, parameters of diastolic function were not assessed in this particular study, but they were assessed in a different study employing a model of T2DM that produces diastolic but not systolic dysfunction [HFD supplementation (60% kcal from lard) for 10-weeks with STZ (75 mg/kg) administered at the 4-week time point]. In this particular study, treatment of male C57BL/6J mice with T2DM with liraglutide during the final 2-weeks (30 μg twice daily) improved diastolic function as indicated by an increased mitral E/A and lower E/e′ ratio (Almutairi et al., 2020). Furthermore, in male C57BL/6 mice subjected to T2DM via HFD supplementation (60% kcal from lard) for 22-weeks with STZ (50 mg/kg) provided for 5-days at the 12-week time point, treatment with exendin-4 (100 μg/kg once daily SQ) during the final 8-weeks improved both diastolic and systolic function as seen by increases in the mitral E/A ratio and LVEF, respectively (Wu et al., 2018). The systolic and diastolic dysfunction were much more severe in the latter study, likely due to increased islet β-cell death and worsened T2DM with higher doses of STZ utilized (treatment for 5 days vs. 1 day). In male Sprague-Dawley rats subjected to T2DM via HFD supplementation for 8-weeks with STZ (35 mg/kg) administered at the 4-week time point, treatment with liraglutide (75 μg once daily) for the final 4-weeks increased both myocardial catalase activity and reduced glutathione levels, while decreasing caspase 3 expression (Hussein et al., 2020). This suggests that GLP-1R agonism attenuates T2DM-related increases in myocardial oxidative stress and cardiac myocyte apoptosis, respectively, key mediators of diabetic cardiomyopathy, though diastolic function was not assessed in this particular study. Nonetheless, treatment with exenatide (24 nmol/kg once daily IP) during the final 4-weeks in mice fed a HFD (45% kcal from lard) for 24-weeks, also attenuated oxidative stress as seen by increases in myocardial catalase and manganese superoxide protein expression, which was associated with an increased mitral E/A ratio (Ding et al., 2019).

In addition to attenuating myocardial oxidative stress and cardiac myocyte apoptosis, other potential mechanisms by which GLP-1R agonism may improve diastolic function and attenuate diabetic cardiomyopathy include an optimization of cardiac energetics. It has been suggested that increases in myocardial glucose oxidation improve cardiac efficiency, which is impaired in T2DM (Lopaschuk et al., 2007), and the liraglutide mediated increase in the mitral E/A ratio and decrease in the E/e′ ratio is associated with increased myocardial pyruvate dehydrogenase activity (Almutairi et al., 2020), the rate-limiting enzyme of glucose oxidation. Systemic liraglutide treatment increased glucose oxidation rates in isolated perfused working hearts from mice with T2DM, but this was not observed following direct treatment of the isolated mouse heart with liraglutide. This is entirely consistent with negligible ventricular GLP-1R expression and suggestive of indirect mechanisms being responsible for these metabolic effects (Ussher and Drucker, 2014; Ussher et al., 2014). These findings are also supportive of decreases in glucose oxidation characterizing the heart in both angiotensin II and phenylephrine infusion mediated diastolic dysfunction in C57BL/6 mice (Mori et al., 2012). Furthermore, cardiac-specific deletion of pyruvate dehydrogenase in mice causes a near complete abolishment of myocardial glucose oxidation rates, which is associated with diastolic dysfunction as indicated by a marked decrease in the mitral E/A ratio (Gopal et al., 2018). Increases in PPARα activity have also been linked to diabetic cardiomyopathy via causing a lipotoxicity associated with increased fatty acid storage and oxidation (Finck et al., 2002), whereas exendin-4 failed to improve the mitral E/A ratio in mice with T2DM and a cardiac-restricted overexpression of PPARα (Wu et al., 2018). Of interest, treatment with liraglutide (30 μg/kg twice daily) during the final week in male C57BL/6 mice fed a HFD (45% kcal from lard) for 32-weeks decreased myocardial protein expression of procollagen 1A1, TNFα, and nuclear localized nuclear factor kappa B (NFκB) (Noyan-Ashraf et al., 2013). While these observations suggest that liraglutide attenuates inflammation and fibrosis, which are key mediators of diabetic cardiomyopathy consistent with the actions of GLP-1R agonists in the setting of ischemic heart disease and heart failure (Ussher and Drucker, 2014; Drucker, 2016), it should be noted that diastolic function was not assessed in this particular study. Due to the limited number of completed clinical and preclinical studies aimed at elucidating the mechanism(s) and extent by which GLP-1R agonists influence diastolic dysfunction in T2DM, further work is clearly necessary to understand the clinical role GLP-1R agonists will play in managing diabetic cardiomyopathy.

## Dipeptidyl Peptidase-4 Inhibitors

DPP-4 inhibitors represent another newer class of antidiabetic therapy that improve glycemia in people with T2DM via augmenting incretin hormone action. As DPP-4 is responsible for the degradation of both GLP-1 and the related incretin hormone, glucose-dependent insulinotropic polypeptide (GIP), DPP-4 inhibitors enhance their bioavailability and thereby lower blood glucose by potentiating islet β-cell insulin secretion in a glucose-dependent manner (Mulvihill and Drucker, 2014). DPP-4 is a widely expressed aminopeptidase that liberates a dipeptide from the N-terminus of various peptides predominantly following a position 2 alanine or proline residue, such as GLP-1 and GIP (Deacon, 2019). Within the myocardium, DPP-4 is primarily expressed in endothelial cells while existing in 2 distinct forms, a soluble circulating form and a membrane-bound form, which exert their effects via both enzymatic and non-enzymatic mechanisms (Mulvihill and Drucker, 2014; Ussher and Drucker, 2014). Despite having the potential to impact whole-body physiology by preventing the degradation of numerous peptide hormones that are DPP-4 substrates (extensively reviewed in Mulvihill and Drucker, 2014), the main driver of glucose lowering in response to DPP-4 inhibition is the ensuing increase in circulating intact GLP-1 and GIP. This was confirmed in sophisticated studies using mice with a genetic deletion for both the GLP-1R and GIP receptor [double incretin receptor knockout (DIRKO) mice]. In response to oral glucose administration, multiple DPP-4 inhibitors (vildagliptin, valine pyrrolidide, SYR106124) have been shown to increase circulating insulin levels and improve glycemia as expected in wild-type littermates, but failed to elicit these actions in DIRKO mice (Hansotia et al., 2004; Flock et al., 2007). Unlike GLP-1R receptor agonists that have been frequently reported to improve cardiovascular outcomes in people with T2DM, DPP-4 inhibitors have not yielded such actions. Reasons for the discrepancy in cardiovascular outcomes between the 2 incretin-based therapies is an important topic of ongoing interrogation currently without an answer. While GLP-1 and GIP are the primary DPP-4 regulated substrates responsible for the glucose lowering actions of DPP-4 inhibitors, many other DPP-4 regulated substrates are cardioactive (e.g., stromal cell-derived factor-1, brain natriuretic peptide, etc.) (Mulvihill and Drucker, 2014). This adds a unique layer of complexity in trying to understand how DPP-4 inhibitors impact cardiac function vs. GLP-1R agonists.

### DPP-4 Inhibitors and Cardiovascular Outcomes

Differing from the GLP-1R agonists, results across the CVOTs completed to date for DPP-4 inhibitors have consistently demonstrated only non-inferiority with respect to the primary composite outcome (3-point MACE). These CVOTs include the Examination of Cardiovascular Outcomes with Alogliptin vs. Standard of Care (EXAMINE), the Saxagliptin Assessment of Vascular Outcomes Recorded in Patients with Diabetes Mellitus–Thrombolysis in MI (SAVOR–TIMI 53), the Trial Evaluating Cardiovascular Outcomes with Sitagliptin (TECOS), and the Cardiovascular Outcomes Study of Linagliptin vs. Glimepiride in Patients with T2D (CAROLINA) (Scirica et al., 2013; White et al., 2013; Green et al., 2015; Rosenstock et al., 2019). Despite a neutral effect on the primary outcome of 3-point MACE, an area of potential concern arising from the completion of DPP-4 inhibitor CVOTs relates to the secondary outcome of rates of hospitalization due to heart failure. Results from SAVOR–TIMI 53 first sparked concern due to the significant increase in hospitalization for heart failure that was observed in patients randomized to saxagliptin vs. placebo (289 vs. 228 events) (Scirica et al., 2013). Although not statistically significant, there were also more events reported in subjects treated with alogliptin or linagliptin for hospitalization for heart failure in the EXAMINE and CAROLINA trials, respectively (85 vs. 79 events in EXAMINE, hazard ratio 1.07; 112 vs. 92 events in CAROLINA, hazard ratio 1.21) (White et al., 2013; Zannad et al., 2015; Rosenstock et al., 2019). On the contrary, rates of hospitalization for heart failure did not differ in subjects with T2DM randomized to sitagliptin in the TECOS CVOT (Green et al., 2015). A number of meta-analyses assessing the overall impact of DPP-4 inhibitors have concluded that as a class, DPP-4 inhibitors do increase the risk of heart failure (Monami et al., 2014; Wu et al., 2014), whereas another meta-analysis concluded that the increase in risk of heart failure is drug-specific, solely attributed to use of saxagliptin (Kongwatcharapong et al., 2016). Mixed results from CVOTs and meta-analyses suggesting a potential drug-specific effect of DPP-4 inhibitors to impact heart failure in people with T2DM, emphasizes the need for future clinical studies to compare different DPP-4 inhibitors head-to-head, while assessing relevant heart failure endpoints in subjects with T2DM and either HFpEF or HFrEF.

### DPP-4 Inhibitors and Diabetic Cardiomyopathy in Humans

Though limited in number, a few smaller human studies have investigated the actions of DPP-4 inhibitors (primarily sitagliptin) on diastolic function (Table 3). Evidence for an improvement in diastolic function was seen in subjects with T2DM inadequately controlled with metformin and glyburide, that were randomized to receive treatment with either sitagliptin (100 mg once daily) or bedtime NPH insulin for 24-weeks (Nogueira et al., 2014). 46.7 vs. 35.7% of subjects experienced a decrease in the E/e′ ratio with sitagliptin vs. NPH insulin treatment, respectively, and this improvement in diastolic function was independent of changes in systolic function and blood pressure. Furthermore, in a randomized, open-label trial of 115 Japanese subjects with T2DM whose blood glucose was inadequately controlled with lifestyle interventions and/or pharmacotherapy, attenuation of increases in the E/e′ ratio was observed with sitagliptin treatment (50 or 100 mg once daily) vs. placebo for 2-years (Yamada et al., 2017). This improvement in diastolic function was once again independent of changes in systolic function and blood pressure, as well as biomarkers of heart failure (e.g., brain natriuretic peptide). In contrast, findings from an open-label, prospective, randomized trial of 80 subjects with T2DM that completed treatment wither either sitagliptin (50 mg once daily) or voglibose (0.6 mg once daily) for 24-weeks, reported no improvement in diastolic function with sitagliptin, as there were no changes in both the e′ and E/e′ ratio (Oe et al., 2015). Of interest, analysis of covariance in these subjects demonstrated that in subjects treated with sitagliptin but not the thiazolidinedione agent, pioglitazone, diastolic function was improved as the e′ increased and the E/e′ ratio decreased. Hence, it remains possible that combination therapy with thiazolidinediones masks potential improvements of diastolic function seen with DPP-4 inhibitor use in subjects with T2DM. In a trial of 174 subjects with T2DM and asymptomatic impaired LV systolic function that completed treatment with either linagliptin (5 mg once daily) or placebo for 48-weeks, ~50% of all subjects exhibited diastolic dysfunction, and linagliptin treatment yielded no changes in e′, as well as the mitral E/A and E/e′ ratios (Cioffi et al., 2020). Based on the available but limited evidence, sitagliptin may produce mild improvements in diastolic function, though further study is necessary, especially with regards to the other approved DPP-4 inhibitors. Because HFpEF is enriched in subjects with T2DM, it will also be important to determine whether the potential increased risk for HF with saxagliptin is specific to either HFpEF or HFrEF, in order to further guide clinical use of this drug class in T2DM associated with heart failure.

**Table 3:** The impact of DPP-4 inhibitors on diabetic cardiomyopathy.

**DPP-4 inhibitor**	**Study model**	**DPP-4 inhibitor mediated outcome**	**References**
Sitagliptin (100 mg once daily)	Subjects with poorly controlled T2DM, on metformin plus glyburide, randomized to receive bedtime NPH insulin or sitagliptin for 24-weeks.	• 46.7 vs. 35.7% of subjects experienced a decrease in the E/e′ ratio with sitagliptin vs. NPH insulin treatment	Nogueira et al., 2014
Sitagliptin (50 or 100 mg once daily)	Subjects with poorly controlled T2DM despite lifestyle interventions and/or pharmacotherapy were randomized to sitagliptin vs. placebo for 2-years	• Decreased E/e′ ratio	Yamada et al., 2017
Sitagliptin (50 mg once daily)	Subjects with T2DM treated with sitagliptin for 24-weeks.	• No changes in both the e′ and E/e′ ratio • Increased e′ and decreased E/e′ ratio in subjects also treated with pioglitazone	Oe et al., 2015
Linagliptin (5 mg once daily)	Subjects with T2DM and asymptomatic impaired LV systolic function treated with linagliptin for 48-weeks.	• No changes in e′ or the mitral E/A and E/e′ ratios	Cioffi et al., 2020
Sitagliptin (16 mg/day in the chow)	Male *db*/*db* mice treated with sitagliptin for 3-weeks	• Reduced relaxation time constant (Tau)	Lenski et al., 2011
Linagliptin (83 mg per kg of chow)	Female C57BL/6J mice supplemented with a high-fat /high sucrose/high-fructose corn syrup diet treated with linagliptin for 4-months.	• Increased e′/a′ ratio • Reduced IVRT • Decreased cardiac interstitial fibrosis	Aroor et al., 2017
Linagliptin (83 mg per kg of chow)	Male Zucker obese rats treated with linagliptin for 8-weeks	• Increase in the e′/a′ ratio • Decrease in the E/e′ ratio, e′, and IVRT	Aroor et al., 2013
MK-0626 (3 mg/kg/day)	Male C57BL/6J mice subjected to T2DM and TAC surgery, treated with MK-0626 for 15-weeks	• Exacerbation of cardiac hypertrophy and reduced LVEF	Mulvihill et al., 2016
Vildagliptin (10 mg/kg/day in the chow)	Male Dahl salt-sensitive rats fed a high-salt diet and treated with vildagliptin for 9-weeks	• Decrease in LVEDP • Increase in the mitral E/A ratio	Nakajima et al., 2019

### DPP-4 Inhibitors and Diabetic Cardiomyopathy in Preclinical Studies

In striking contrast to observations from clinical studies of DPP-4 inhibitors, preclinical studies have frequently demonstrated that use of DPP-4 inhibitors confers cardioprotection, especially in the setting of MI or HFrEF (Mulvihill and Drucker, 2014; Ussher and Drucker, 2014; Remm et al., 2016). This cardioprotection appears to extend to diastolic dysfunction and HFpEF as well, as sitagliptin treatment for 3-weeks (provided in the diet at 16 mg/day) in 6-week-old male *db*/*db* mice resulted in a reduction in the relaxation time constant (Tau) assessed using high fidelity conductance catheters (Lenski et al., 2011). Similarly, a 4-month treatment with linagliptin (provided in the diet at 83 mg per kg of chow) in 4-week-old female C57BL/6J mice supplemented with a high-fat (46% kcal)/high sucrose (17.5% kcal)/high-fructose corn syrup (17.5%) diet, improved diastolic function as indicated by an increased e′/a′ ratio and reduced IVRT (Aroor et al., 2017). Furthermore, picro-sirius red staining revealed that linagliptin treatment also prevented the 2-fold increase in cardiac interstitial fibrosis observed in control treated mice. Likewise, 8-weeks of treatment with linagliptin (provided in the diet at 83 mg per kg of chow) improved diastolic function in male Zucker obese rats as seen by an increase in the e′/a′ ratio, as well as a decrease in the E/e′ ratio and IVRT (Aroor et al., 2013). Conversely, in male C57BL/6J mice subjected to T2DM via HFD supplementation (45% kcal from lard) for 28-weeks with STZ (90 mg/kg) administered and TAC surgery performed at the 12-week and 18-week time points, respectively, treatment with the DPP-4 inhibitor, MK-0626 (provided in the diet to achieve 3 mg/kg/day), for the final 15-weeks exacerbated cardiac hypertrophy and reduced LVEF (Mulvihill et al., 2016). As mentioned previously though, parameters of diastolic function were not assessed in this particular study, but mice treated with MK-0626 demonstrated elevations in mRNA expression for markers of fibrosis and inflammation, both of which are proposed mediators of diastolic dysfunction (Jia et al., 2018; Ritchie and Abel, 2020). Interestingly, DPP-4 inhibition with MK-0626 (provided in the diet to achieve 3 mg/kg/day) for 4-weeks produces opposing actions in lean male C57BL/6 mice, whereby the decline in LV fractional shortening and induction of cardiac fibrosis assessed with Masson's trichrome staining were attenuated at 4-weeks post-TAC, though diastolic function was again not assessed (Hirose et al., 2017). In 11-week-old Dahl salt-sensitive rats fed a high-salt diet (8.0% NaCl) to induce HFpEF, treatment with vildagliptin (provided in the drinking water to achieve 10 mg/kg/day) alleviated diastolic dysfunction as indicated by a decrease in LVEDP and an increase in the mitral E/A ratio (Nakajima et al., 2019). Nonetheless, Dahl salt-sensitive rats with HFpEF exhibit decreases in body weight, and it remains undetermined whether such improvements in diastolic function would still be observed in the presence of underlying obesity/T2DM in this model.

Taken together, various DPP-4 inhibitors do appear to improve indices of diastolic function in various experimental models of either T2DM or heart failure (Table 3), though it does appear that the impact on heart failure pathogenesis can be influenced by the underlying presence of obesity, insulin resistance and metabolic dysfunction. While reductions in cardiac fibrosis are consistently reported and may contribute to how DPP-4 inhibition improves diastolic function in preclinical studies (Lenski et al., 2011; Aroor et al., 2017; Hirose et al., 2017; Nakajima et al., 2019), other potential mechanisms at play include reductions in cardiac lipotoxicity due to reduced protein expression of the fatty acid transporter, CD36 (Lenski et al., 2011). Moreover, the linagliptin mediated improvement of diastolic function in high-fat/high sucrose/high-fructose fed female C57BL/6J mice may involve restricting TRAF3 interacting protein-induced inflammation, a cytoplasmic adapter molecule that acts as an upstream regulator of NFκB (Aroor et al., 2017). Continuing with inflammation, direct treatment of H9c2 myoblasts with sitagliptin (0, 0.1–4 μM) prevented lipopolysaccharide (10 μg/mL)-induced increases in mRNA/protein expression of key inflammatory mediators such as TNFα and interleukin-6, while also reducing nuclear NFκB levels (Lin and Lin, 2016). Whether H9c2 myoblasts express DPP-4 has not been conclusively determined though, and such findings suggest that direct DPP-4 inhibition *per se* is sufficient to reduce cardiac myocyte inflammation independent of increases in circulating DPP-4 substrates that can transduce receptor-mediated signaling in the myocardium. It should be noted that most of these observations have simply been associations, with no extensive interrogation performed to determine whether these factors are causally related to the actual improvement in diastolic function in response to DPP-4 inhibition. As previously alluded to, many DPP-4 substrates are also cardioactive, including GLP-1, GIP, stromal cell-derived factor-1, and brain natriuretic peptide to name a few (Mulvihill and Drucker, 2014; Ussher and Drucker, 2014; Greenwell et al., 2020). Hence, it is likely that multiple signaling pathways engaged by these substrates, and potentially disengaged due to reduction in the formation of bioactive breakdown products [e.g., GLP-1(9-36)], will make it difficult to intricately tease out the mechanisms by which DPP-4 inhibitors regulate diastolic function.

## Final Summary, Potential Caveats and Future Directions

The completion of multiple CVOTs for SGLT2 inhibitors and incretin-based therapies has indeed invigorated excitement in the field of cardiovascular endocrinology, as we now have data in tens of thousands of subjects with T2DM and high risk cardiovascular disease treated with these agents. As such, SGLT2 inhibitors and GLP-1R agonists to an extent, have been shown to reduce cardiovascular events in people with T2DM, while also reducing hospitalization rates for heart failure. On the other hand, DPP-4 inhibitors for most part have been shown to be non-inferior to standard of care with regards to these outcomes in people with T2DM. However, these CVOTs were not designed to evaluate key parameters of cardiac function or to evaluate relevant endpoints in subjects also comorbid for MI or HF, nor were they designed to separately evaluate T2DM subjects with either HFrEF of HFpEF. Because HFpEF is often enriched in the T2DM population, while many subjects with T2DM often exhibit a subclinical asymptomatic stage of diabetic cardiomyopathy characterized by diastolic dysfunction, it is imperative that future studies elucidate the differing actions of these therapies on systolic and diastolic function. Advances in this area will undoubtedly play a major role in therapeutic decision making with regards to managing subjects with diabetic cardiomyopathy.

In order to better understand how SGLT2 inhibitors and incretin-based therapies influence diastolic function in T2DM, while elucidating the mechanisms involved, continued preclinical studies utilizing both pharmacological and mouse genetics approaches are necessary. It should be noted though that preclinical studies often do not include comparator groups to account for glucose lowering, weight loss, or other lifestyle interventions (e.g., exercise) that are likely relevant to subjects with T2DM on these medications, and all capable of affecting diastolic function. It will be essential for future preclinical studies to also elucidate how these other factors may influence diastolic function either synergistically or negatively when combined with SGLT2 inhibitors or incretin-based therapies, in order to understand the true translational potential of these therapies specifically for diabetic cardiomyopathy. Importantly, we share the viewpoint of others (Ritchie and Abel, 2020) that perhaps now would be the most opportune time to redefine diabetic cardiomyopathy, and that diabetic heart disease might be the more fitting term to reflect a disorder that is more prominent in subjects with T2DM than initially realized. It also remains possible that none of the current therapies will prove truly effective for attenuating diastolic dysfunction in subjects with T2DM. Thus, it is also necessary we continue to identify the molecular mediators responsible for the pathology of diabetic cardiomyopathy and/or T2DM-related diastolic dysfunction, as these may represent new targets to pursue for the specific treatment of diabetic heart disease.

## Author Contributions

All authors researched literature, drafted and wrote the review article, and approved the submitted version.

## Conflict of Interest

The authors declare that the research was conducted in the absence of any commercial or financial relationships that could be construed as a potential conflict of interest.
